# Comparative Analysis of Examination Methods for Periapical Lesion Diagnostics: Assessing Cone-Beam Computer Tomography, Ultrasound, and Periapical Radiography

**DOI:** 10.3390/diagnostics14070766

**Published:** 2024-04-04

**Authors:** Aleksandra Karkle, Anda Slaidina, Maksims Zolovs, Anete Vaskevica, Dita Meistere, Zanda Bokvalde, Laura Neimane

**Affiliations:** 1 Department of Conservative Dentistry and Oral Health, Riga Stradins University, LV-1007 Riga, Latvia; anete.vaskevica@rsu.lv (A.V.); zanda.bokvalde@rsu.lv (Z.B.);; 2 RSU Institute of Stomatology, LV-1007 Riga, Latvia; anda.slaidina@rsu.lv; 3 Department of Prosthetic Dentistry, Riga Stradins University, LV-1007 Riga, Latvia; 4 Statistics Unit, Riga Stradins University, LV-1007 Riga, Latvia; maksims.zolovs@rsu.lv; 5 Institute of Life Sciences and Technology, Daugavpils University, LV-5401 Daugavpils, Latvia

**Keywords:** apical surgery, cone-beam computer tomography radiographs, endodontics, periapical lesion, periapical X-ray, ultrasonography

## Abstract

Introduction: Periapical lesions of teeth are typically evaluated using periapical X-rays (PA) or cone-beam computer tomography (CBCT); however, ultrasound imaging (US) can also be used to detect bone defects. A comparative analysis is necessary to establish the diagnostic accuracy of US for the detection of periapical lesions in comparison with PA and CBCT. Objectives: This study aimed to evaluate and compare the measurement precision of US against PA and CBCT in detecting periapical lesions. Methods: This study included 43 maxillary and mandibular teeth with periapical lesions. All teeth were examined clinically, radiographically, and ultrasonographically. Observers evaluated and measured the periapical lesions on CBCT, PA, and US images. Results: The comparison of lesion size showed that it differs significantly between the different methods of examination. A statistically significant difference was found between CBCT and US (mean difference = 0.99 mm, 95% CI [0.43–1.55]), as well as between CBCT and PA (mean difference = 0.61 mm, 95% CI [0.17–1.05]). No difference was found between the US and PA methods (*p* = 0.193). Conclusion: US cannot replace PA radiography in detecting pathologies but it can accurately measure and characterize periapical lesions with minimal radiation exposure. CBCT is the most precise and radiation-intensive method so it should only be used for complex cases.

## 1. Introduction

A periapical lesion is a local inflammatory response of bone around the apical part of the tooth, which may be formed by bacterial penetration from the dental pulp following pulp necrosis and periodontal disease [1]. Bone defects and periapical lesions are closely related to microbial infection and inflammatory processes management, bone resorption, and structural changes in the periapical area. In most cases, successful endodontic treatment promotes the healing of apical pathology and ensures the recovery process but in some cases, periapical lesions may be unrelated to the tooth and might be of different origin, for instance, trauma-induced lesions. The importance of accuracy in periapical lesion diagnostics cannot be overstated. Accurate diagnosis is essential for effective treatment planning and the successful management of the condition [2] as well as determining the correct size of the lesion. Evaluation of the patient requires not only gathering a complete medical and dental history, performing a thorough dental examination, and evaluating the patient’s emotional condition and expectations [1] but also a high-quality radiological investigation. 

Radiological investigations and various imaging techniques have been provided to help dentists discover, evaluate, and diagnose odontogenic periapical lesions and plan and conduct follow-up procedures [2]. They include cone-beam computer tomography (CBCT), panoramic radiographs (PAN), conventional periapical (PA) radiographs, magnetic resonance imaging (MRI), and ultrasound imaging (US) [3].

CBCT and periapical PA X-ray are two of the most used dental imaging methods to diagnose dental periapical lesions. PA radiographs are the foremost commonly accessible instrument within the dental clinic due to the availability and costs of investigative methods. PA X-rays provide two-dimensional information about the teeth and surrounding tissue in the patient’s jaw. This diagnostic method has been used to recognize specific dental problems such as tooth decay, root canal infection, and periapical lesions [3,4,5]. The downside of this method is that it is two-dimensional, and it is frequently not possible to evaluate the true size of the apical lesion. The American Association of Endodontists’ (AAE) guidelines for PA X-rays in routine or emergency oral health check-ups typically cover several key aspects, including patient selection, imaging technique, positioning, and investigation results interpretation. PA X-rays need to be performed when clinical examination suggests dental caries, pulpal or periapical pathology, periapical lesions, or dental trauma. The AAE report that PA X-rays are crucial in detecting, diagnosing, and assessing the periapical lesions such as abscesses, granulomas, cysts, and other pathologies associated with the tooth root and surrounding anatomical structures [6]. High-quality digital X-ray radiography should also be used for enhanced image quality, lower radiation dose, and ease of storage and retrieval [7]. At present, CBCT is increasingly being used to diagnose periapical lesions in complicated cases. The AAE provides guidelines for the use of CBCT in cases where two-dimensional radiographs are insufficient to provide comprehensive information about lesion characteristics, magnitude, location, size, proximity to vital anatomical structures, and potential treatment complications. Additionally, the AAE recommends that clinicians consider the patient’s clinical examination and medical history to evaluate the specific diagnostic needs intraoral radiography cannot meet [4,5]. CBCT examination provides a broad overview of the investigated field and is used for more complex dental procedures, such as oral surgery, implant placement, orthodontics or endodontic retreatments, and endodontics surgery [6,8,9,10]. CBCT, deemed the “gold standard” for diagnosing odontogenic pathologies, offers superior precision compared to PA radiographs. CBCT provides detailed images and valuable diagnostic information but it is often more costly than PA radiography in terms of the equipment and operating costs. Three-dimensional imaging incurs higher costs from purchasing specialized equipment and software licensing as well as maintenance and operator training. These expenses influence the cost of dental services as clinics aim to recover these investments and for this reason, the financial burden extends to both healthcare facilities and patients [11]. Comparing CBCT to traditional CT scans, radiation doses are generally lower; however, comparing the radiation dose between PA X-ray, PAN, and CBCT, above all, a three-dimensional examination will require a larger radiation dosage than a two-dimensional examination. As outlined in the guidelines by the European Academy of Dento-Maxillofacial Radiology, the recommended effective dose for a single intraoral radiograph is 1.5 μSv. Furthermore, the effective dose for panoramic radiography is around 22.0 μSv, while for CBCT examination, it ranges from 61 to 134 μSv [12]. CBCT scans often entail a radiation dose 3 to 6 times higher than a PAN, depending on the mode used, imaging protocols, machine settings, and the area scanned—be it a section, fragment, or the full jaw. Many CBCT devices integrate safety measures like collimation and customizable exposure settings to mitigate radiation exposure [13]. Due to the higher radiation doses involved, current research does not support its regular use for diagnostic purposes [14]. This is particularly important for children and adolescents as they are more susceptible to the potential risks associated with ionizing radiation. Therefore, it is crucial to consider measures aimed at reducing radiation exposure in these patient groups [15]. In addition, proper training and experience are essential for clinicians to interpret CBCT images accurately. It is necessary to assess precisely the extent and cause of the lesion, determine the best course of action for therapy, and lower the risk of potential complications. Moreover, all clinicians using CBCT must have the appropriate accredited training. Dental undergraduate and endodontic post-graduate programs should incorporate CBCT-related education into their curricula, such as the mode of operation, justification, interpretation, and reporting of CBCT images [16]. Other techniques, such as US, can also be used to differentiate periapical lesions [5,17,18]. US is a painless, non-ionizing radiation imaging method that uses high-energy sound waves to examine internal human tissues and organs. On a screen, the sound waves’ echoes create images of the investigated area. Currently, US is becoming increasingly utilized for imaging the maxillofacial area, gaining widespread acceptance as a diagnostic tool due to its numerous benefits [19,20,21]. It is employed for detecting various conditions, such as fractures in the mid-facial region, ramus, and condyle, swellings in the head and neck, and disorders of the temporomandibular joint, salivary glands, and cervical lymph nodes. Additionally, US can be used to identify intraosseous lesions, such as periapical lesions, and provide valuable information on blood flow direction using Doppler technology. Combining these features, US contributes significantly to precise lesion diagnosis and aids in developing appropriate treatment plans [5,22,23]. US does not present the same risks as radiological evaluations and can be successfully used in dentistry and oral surgery to image periapical bone defects and lesions [20,24]. This study was conducted with the aim of determining the ability of US to detect periapical lesions and compare the precision of US measurements with PA X-ray and CBCT. 

## 2. Materials and Methods

### 2.1. Study Subjects

This cross-sectional study included 34 patients of both genders aged from 18 to 78 (mean age: 43.1 ± 14.6 y) who were referred to the Department of Endodontics, Riga Stradins University Institute of Stomatology (Riga, Latvia) for endodontic apical surgery. The period from September 2021 to May 2023 was selected for the study, and it involved 43 teeth with periapical lesions associated with maxillary and mandibular incisors, canines, and premolars. The study was conducted in accordance with the Declaration of Helsinki and approved by the Ethics Committee of Riga Stradins University (protocol Nr. 22-2/427/2021; date of approval: 11 August 2021). Prior to participation, patients were provided with comprehensive information regarding the study’s aim, design, procedures, and protocols, underscoring the commitment to ethical research conduct. After providing informed consent, the individuals were included in the study. 

### 2.2. Inclusion and Exclusion Criteria

The inclusion criteria for this study were adult patients with precisely defined periapical lesions related to maxillary or mandibular incisors and premolar teeth as a sequel to persistent endodontic infection after root canal treatment/retreatment with/without the sinus tract, root perforations; cases deemed unsuitable for non-surgical endodontic intervention; or cases with trauma clearly indicated for endodontic apical surgery. We excluded patients with lesions unrelated to the root apical area; in possession of vital teeth with radiolucency in the apical region; that were pregnant; younger than 18 years old; with non-restorable teeth; advanced periodontal disease, including endo-perio lesions; uncontrolled systemic health conditions; receiving bisphosphonate therapy; receiving orthodontic treatment. Both jaw molar teeth were excluded from the study as intraoral US would be burdensome due to difficult access and interference from anatomical structures.

### 2.3. Examination

A specific form was created to document the records of the patient’s medical history, and clinical examinations of the tooth, including endodontic tests, pulp vitality, percussion, palpation, pocket measuring, and mobility tests, were carried out. All patients included in the study were scheduled for apical surgery procedures; therefore, in essence, each patient was examined with PA X-ray, CBCT—as required before apical surgery—and US imaging. 

#### 2.3.1. Periapical (PA) Radiography Examination and Evaluation

For the PA X-ray procedure, all patients were positioned properly to ensure accurate and clear images and were provided with a protective shield to guard against radiation exposure. PA radiographs were taken by using a paralleling technique, utilizing a Durr Imaging Plate and Film Holder System (Durr Dental, Bietigheim-Bissingen, Germany) with intraoral phosphor sensor plates (Durr Dental, Bietigheim-Bissingen, GermanyBietigheim-Bissingen) and a Planmeca Prox dental X-ray unit (Planmeca, Helsinki, Finland). VistaSoft software 3.0.31 was used for image interpretation. If necessary, different image processing facilities provided by the VistasScan software 3.0.31 system were used to ensure accuracy.

For periapical lesion detection, three scale classification categories were used [21]:

Category 1: no discernible periapical lesion was identified;
Category 2: a periapical lesion was possibly present, however, the distinction was not unequivocal;
Category 3: a periapical lesion was definitively observed.

**Figure 1:**
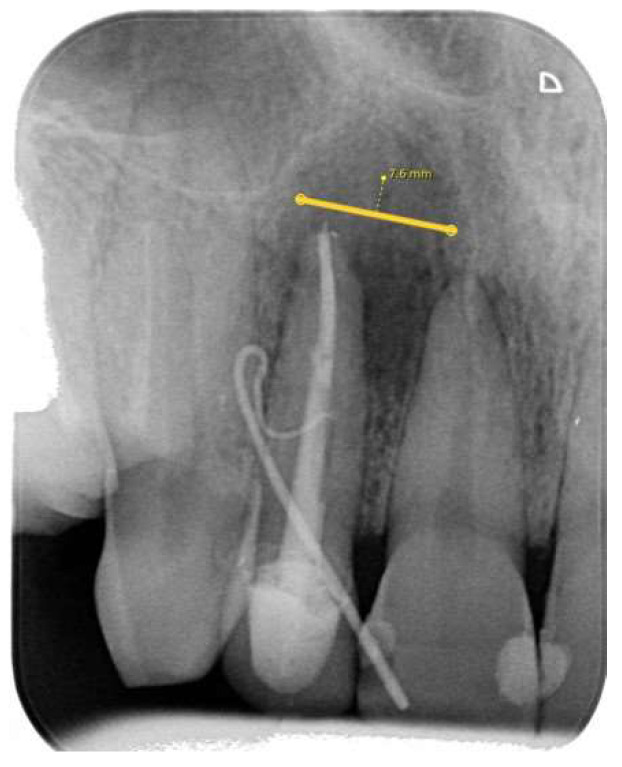
PA radiograph showing the measurement of a periapical lesion related to the maxillary right second incisor.

In addition, every lesion’s size was measured in millimeters (mm) at the widest periapical lesion area perpendicular to the tooth apex in the mesio–distal (MD) direction using a digital ruler (Figure 1).

#### 2.3.2. Cone-Beam Computer Tomography (CBCT) Radiographic Examination and Evaluation

CBCT scans were acquired using the same protocol on a single unit (Veraview 800X, Morita, Japan) operating at 102 kV, 5.1 mA. The field of view (FOV) was set at 40 × 40 × 40 mm with a voxel size of 0.125 mm. To ensure optimal viewing conditions, i-Dixel software Version 2, 15, 0 (Morita, Osaka, Japan) was used for image analysis. 

**Figure 2:**
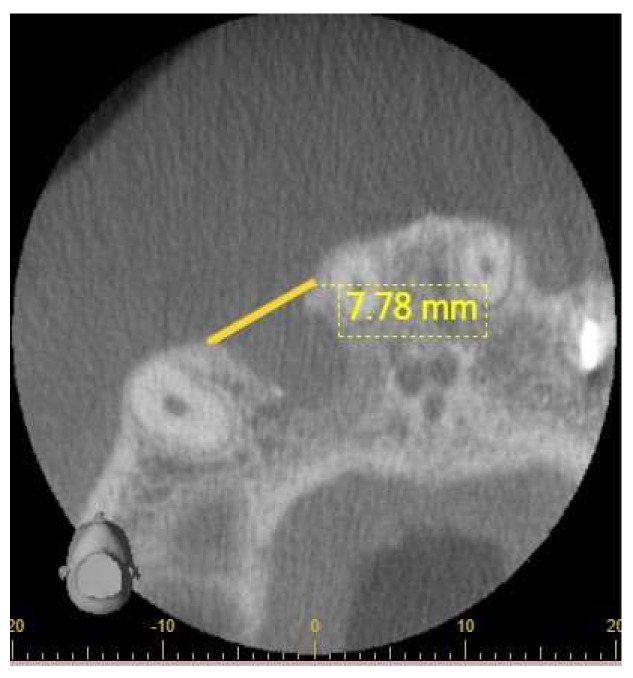
Periapical lesion measured on a CBCT image in mm in the MD direction.

CBCT images were analyzed in the axial view assessed to precisely measure lesion dimensions in millimeters in the MD widest direction (Figure 2). Lesion detection employed a three-scale classification system for assessing periapical lesions’ absence, presence, or possible presence. This assessment aligns with the classification approaches used in PA radiography and US examinations. CBCT software Version 2, 15, 0 tools were used to navigate through different planes, including axial, coronal, and sagittal views. These views offer different perspectives on the area of interest. The axial plane view was chosen to identify the widest cortical bone disruption and lesion size measurement because it provides a horizontal cross-section of the area, which is helpful for assessing the extent of the periapical lesion and any associated cortical bone disruption. Once the widest point was identified, the measurement tool integrated into the respective software was employed. Image processing facilities provided by the i-Dixel software Version 2, 15, 0 system were used to allow the observers to improve the image quality. This helps quantify the extent of the disruption and provides valuable information for treatment planning. 

The Dell 380 Precision workstation, in conjunction with an 18-inch LCD Dell monitor boasting a resolution of 1024 × 768 pixels (Dell Computer Corporation, Round Rock, TX, USA), was used for the determination of periapical lesion presence or absence and size measurements in the PA X-ray and CBCT images.

#### 2.3.3. Ultrasound Examination and Evaluation

**Figure 3:**
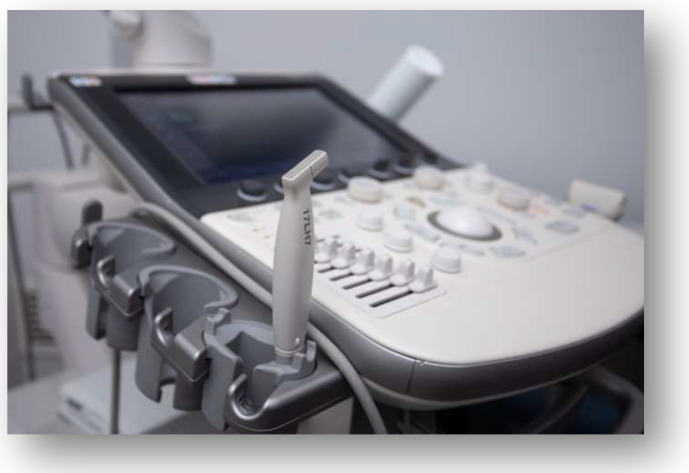
A high-frequency ‘hockey stick’ linear transducer.

The US investigation was performed by an experienced oral radiologist. Examinations were conducted using the Apolio a450, a Canon ultrasound machine (Canon Medical Systems Corporation, Shimoishigami Otawara, Tochigi, Japan). A high-frequency hockey stick linear transducer with a 20-millimeter wide scan (frequency 15–18 MHz) was used (Figure 3). 

**Figure 4:**
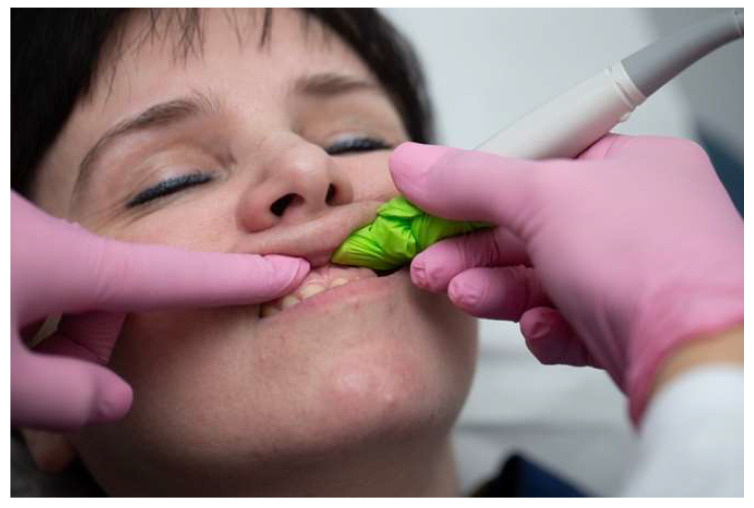
Intraoral US transverse scan.

**Figure 5:**
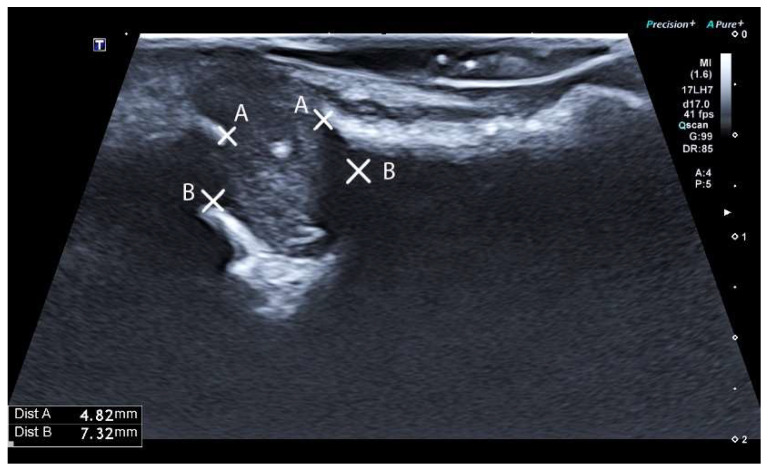
US image of the bone defect.

The transducer was coated with ultrasound gel (Clinical Ultrasound gel, Diagramm Halbach, Schwerte, Germany) and covered with a single-use covering. It was carefully positioned intraorally relative to the apical region of the tooth under investigation. The transducer was slowly maneuvered to delineate the bone defect. Upon identifying a defect, images were captured. The transducer position was moved and modified multiple times to obtain an adequate number of sufficient images. Every lesion was assessed and recorded in the MD plane (Figure 4). The US image shows that the periapical lesion gives the impression of being a dark, hypoechoic area around the apical part of the tooth. It is presented as a well-defined or irregularly shaped region depending on the nature and field of the periapical lesion. The presence of inflammation, infection, or pathological changes in the periapical area indicates the previously mentioned dark area. The US image shows discontinuity in the bone structure as irregular or fragmented bone edges or even the complete absence of bone in several cases (Figure 5). In the absence of a recognized standard for US classification of periapical lesions in the current literature, classification criteria analogous to those used in PA radiographic and CBCT assessments were adopted for ultrasound evaluation.

A three-scale classification system was implemented to determine the possible presence, definitive presence, or absence of periapical lesions.

The determination of periapical lesion presence or absence and size measurements in the US images was carried out using the Apolio a450 (Canon Medical Systems Corporation, Shimoishigami Otawara, Tochigi, Japan), a Canon ultrasound machine digital measurement tool. 

All PA X-ray, CBCT, and US images were assessed by two observers (oral radiologist Z.B. with 5 years of experience and endodontist A.K. with 5 years of experience). 

### 2.4. Statistical Analysis

Since no pilot study was conducted and no suitable data were found in the studies available in the literature, the necessary sample sizes were generally calculated. A priori sample size calculation was performed using GPower (v. 3.1.9.7) [25]. The following parameters were specified: α = 0.05 (significance level), power = 0.90 (desired probability of detecting a true effect), effect size = 0.25 (considered a moderate effect size according to [26]) number of measurements = 3 (repeated measures ANOVA with within-subject factors). Based on these parameters, GPower recommended a minimum sample size of *n* = 36.

To compare the examination methods (PA X-ray, US, and CBCT), a one-way repeated measures ANOVA was conducted because this study design involves multiple measures of the same variable (periapical lesions size measured in millimeters) taken from the same subjects (patients) under different conditions (examination methods: PA X-ray, US, and CBCT). The assumption of data distribution was assessed through inspection of normal Q–Q plots and Shapiro–Wilk test results, and the assumption of sphericity was tested using Mauchly’s test. Generalized estimating equations (GEEs) were used to compare repeated measures of periapical lesions measured in scores between the PA X-ray, US, and CBCT methods. The overall performance and sensitivity of the examination methods (PA X-ray, US, and CBCT) in detecting periapical lesions were tested with dichotomous 2 × 2 tables. It was assumed that the tooth had a periapical lesion if it was definitively observed (Category 3) with the examination method used. The ground truth was assumed to be that all teeth included in the study had a periapical lesion. 

The observers’ agreement regarding the qualitative evaluation of periapical lesions was assessed via Cohen’s kappa coefficient, whereas quantitative evaluation was assessed via the interclass correlation coefficient.

## 3. Results

The most examined teeth group in the study was the maxillary incisors, which made up 68% of the total examined teeth. In comparison, the mandibular incisors accounted for 14% of the teeth that were assessed. However, there was relatively less emphasis on the assessment of the maxillary and mandibular canines and premolars, which together constituted only 18% of the examination (Table 1).

### 3.1. Comparison of Examination Methods

**Figure 6:**
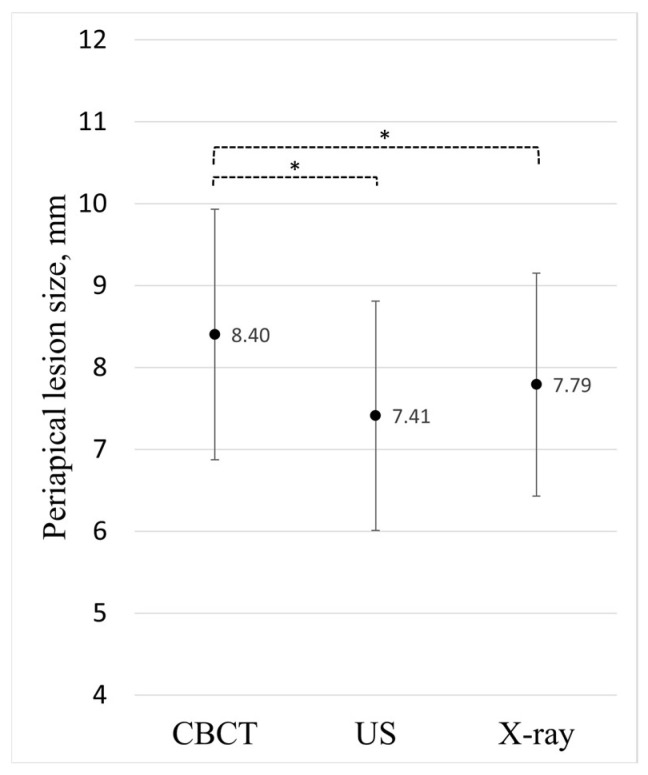
Comparison of periapical lesion sizes. CBCT, US, and PA X-ray examinations for the same individuals. Means with a 95% confidence interval connected and marked with an asterisk show a statistically significant difference between the examination methods (*p* < 0.001, η^2^_G_ = 0.008 (low effect size)).

The comparison of periapical lesion size showed that it differs significantly between the methods of examination: F (2, 84) = 8.61, *p* < 0.001, η^2^_G_ = 0.008 (low effect size). A statistically significant difference was found between CBCT and US (mean difference = 0.99 mm, 95% CI [0.43–1.55]), as well as between CBCT and PA X-ray (mean difference = 0.61 mm, 95% CI [0.17–1.05]), whereas no difference was found between the US and PA X-ray methods (*p* = 0.193) (Figure 6).

The results of the GEE analysis showed significant differences in periapical lesion scores between methods (*p* < 0.001), whereby the US method had a significantly lower score on average, 0.34, compared to CBCT. No other differences were observed (*p* > 0.05).

The highest sensitivity to detect periapical lesions was observed for CBCT (97.7%), followed by PA X-ray (93.0%), while US exhibited the lowest level of sensitivity of 76.7%.

### 3.2. Observers’ Agreement: Assessing Periapical Lesions through Qualitative and Quantitative Evaluation

Qualitative evaluation showed that the calculated Cohen’s kappa coefficient was 0.48 (PA X-ray), 0.67 (US), and 1.0 (CBCT), indicating agreement between Observer 1 and Observer 2 beyond chance (*p* < 0.001). According to Landis and Koch’s interpretation scale, these kappa values fall within the category of moderate (PA X-ray), substantial (US), and perfect (CBCT) agreement. 

**Figure 7:**
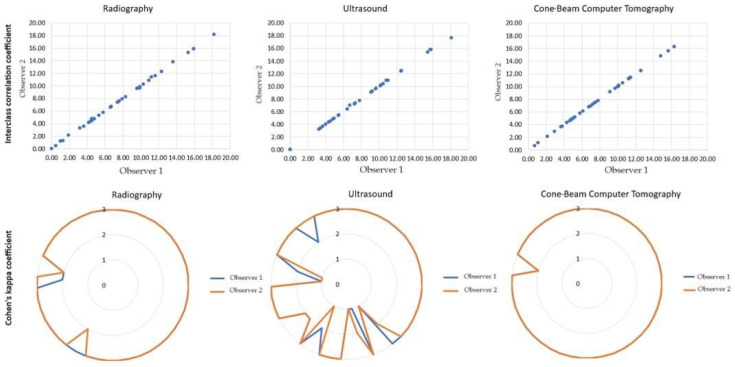
Interclass correlation coefficient and Cohen’s kappa coefficient results showing reliability between observers.

The quantitative evaluation showed that the interclass correlation coefficient of PA X-ray, US, and CBCT was 1.0, 95% [1.0–1.0], *p* < 0.001, indicating perfect agreement between observers (Figure 7).

## 4. Discussion

PA X-ray has been used in dentistry for more than a century for diagnostic purposes for conditions that cannot be assessed clinically [27]. PA X-ray is mainly used because of its availability, simplicity, and low cost [28]. Although PA X-rays provide a two-dimensional image of a three-dimensional object, this in turn causes limitations in assessing the anatomical structure and surrounding lesion’s diagnostic accuracy [20]. In addition, the connections between soft and hard tissues are not visible on PA radiographs. The two-dimensional radiographic method can be used to visualize objects in the apical–coronal and buccal–lingual planes but the buco–lingual plane cannot be defined [29]. Therefore, a smaller bone destruction size is depicted on the radiograph than is truly present. Nevertheless, PA X-ray is the most used imaging technique to determine and evaluate periapical lesions in dentistry.

The use of CBCT in dentistry was first mentioned in 1998 [30] when a CBCT image was created using a 2D detector with a cone-shaped beam form, which performs one rotation around the object. As a result, a series of 2D images are obtained, which are reconstructed in 3D using a modification of the original cone-beam algorithm [31]. Therefore, the advantage of CBCT is the method’s accuracy and informativeness, especially in complex cases because of its ability to detect various lesions, tumors, traumatic injuries, and maxillofacial developmental anomalies. It also allows for a clearer visualization of tooth root structure, canal anatomy, resorptions, as well as various other small details. However, CBCT should only be used judiciously to avoid exposing patients to ionizing radiation unnecessarily in cases where it would not improve treatment planning or diagnostic accuracy [28]. The quality of this diagnostic method can be affected by various factors, the most common of which is the movement of the patient during the examination. This can contribute to diagnostic difficulties and the acquisition of low-quality images. In cases where re-examination would be necessary due to the patient’s movement, all risks associated with radiation dose for the patient must be evaluated beforehand. The image quality also depends on the materials of various dental artifacts, which can influence image quality and diagnosis accuracy [32]. All practitioners utilizing CBCT must undergo accredited training. Dental undergraduate and post-graduate endodontic programs should include education on CBCT, covering aspects such as its operation, rationale for use, interpretation, and the reporting of CBCT images [16].

Ionizing radiation investigation methods, such as PA radiographs and CBCT, have been reliable methods used in dental radiography since the invention of the X-ray. With the beginning of the era of digitalization, radiation doses have decreased; however, the risks of exposure are still present [33]. The radiation dosages that patients are exposed to during dental X-rays are relatively modest; nevertheless, proof of an increased risk of cancer from modest doses of radiation is well documented in numerous studies [34]. Repeated exposure may also increase the risk of developing cancer [35]. There are studies that discuss cancer risk in the head and neck regions related to exposure to dental X-rays [36,37].

The US investigation method was introduced into medical practice during World War II [19]. As a non-invasive, affordable method that has an advantage over other conventional radiography modalities by removing the negative biological consequences of X-ray emission, it is an essential instrument in radiological diagnosis. At present, US imaging investigations are used in various medical applications, including cardiology, obstetrics, gastroenterology, urology, and musculoskeletal imaging. However, in the dento-facial region, US is not frequently used. This is mainly because US is a soft tissue diagnostic method. However, the capability to perform dynamic diagnostics in real-time is a significant benefit, granting clinicians the ability to assess the function and characteristics of soft tissues, which is less impacted by the patient’s positioning during the examination [38].

Ultrasound in the dento-facial region has the potential to offer detailed images of structures such as the tongue, salivary glands, temporomandibular joint (TMJ), and periapical lesions. This imaging modality can provide valuable insights into conditions such as cysts, tumors, and salivary gland disorders. Previous studies have shown that US images of jaw lesions are feasible because of the potential for fenestration and the thin anterior bone. Still, the inconvenience of the transducer size and extra-oral position was cited as one of the weaknesses of the investigation method [39]. The US intraoral ‘hockey stick’ linear transducer provides an opportunity for examining physicians to carry out a more accurate examination. Compared to the external transducer, the small size and shape of the intraoral transducer make it easier to orientate the lesion in the mouth in different regions without using the radiograph for reference. Transverse and longitudinal scans can be acquired by inserting the ultrasonic probe intraorally into the buccal sulcus above the apical region of the involved tooth. However, intraoral scanning is not feasible if the patient’s vestibule is too shallow, the cortical bone level is thick, or defects are located away from the palatal side because of transducer placement options. In some cases, the canine region can only partially be seen on the US image because of the root length and its localization. However, during US investigation, if the periapical lesion was detected, lesion size could be measured precisely. Another potential setback could be due to the lack of skill of the specialist performing the manipulation. The effectiveness of ultrasound relies heavily on the skill of the operator, particularly in dental and maxillofacial applications where the anatomy is complex and the need for high-level expertise in both performing the procedure and interpreting the results is paramount [40]. This need for specialized training represents the main challenge to its widespread adoption in dental practices, where resources for such training may be limited and the need for clear visualization of hard tissues, which ultrasonography cannot provide as effectively as other modalities, is often critical. Training and calibration of the operators are essential, particularly considering the many systems available and the notable progress made in periapical lesion imaging. At present, a trained and experienced physician can employ multi-modular examinations to obtain the data required regarding periapical lesions [39].

The results obtained in this study revealed that there is a dubious difference between the examination methods, namely, CBCT, US, and PA X-ray, in detecting periapical lesions. Whereas CBCT exhibited a statistically significant difference in detecting and measuring the size of the periapical lesion compared to the other methods, the associated effect size coefficient was relatively small, indicating low practical relevance. 

No difference was found between the US and PA X-ray imaging methods in measurement precision. Meanwhile, PA X-ray involves lower costs and is much more accessible than US. Although PA X-ray presents low radiation risks, of these two modalities, the latter would evidently be more suitable for the diagnosis of periapical lesions; however, unlike X-rays, US does not involve ionizing radiation, which enhances safety, especially for frequent examinations in sensitive groups such as young adults and children. Secondly, US enables the real-time imaging and dynamic evaluation of soft tissues, providing valuable insights into tissue vascularity and fluid dynamics [41].

Compared to CBCT, PA X-ray is cheaper, easier to access, more familiar to the dental specialist, and subjects the patient to a much lower radiation dose, suggesting that it is the method of choice in daily practice; however, if a definitive diagnosis with detailed information about the size, origin, and extent of the lesion is crucial, obtaining three-dimensional information with CBCT is justified. In addition, CBCT scans are typically simpler for patients to understand than those for PA X-rays because they offer a clear, three-dimensional view of their oral structure. This aids patients in gaining a better understanding of their diagnosis and potential treatment choices [32]. The American Academy of Oral and Maxillofacial Radiology (AAOMR) and the AAE have developed guidelines with evidence-based recommendations for CBCT use in endodontic case management [42]. In addition, the European Society of Endodontology (ESE) and the European Commission Directorate–General for Energy have represented an evidence-based consensus of an expert committee convened on the use of CBCT in endodontics [43,44].

The use of CBCT imaging is recommended for select clinical cases that will benefit from additional information. Selective applications of CBCT imaging are relevant since reducing unnecessary imaging avoids unnecessary patient radiation exposure and decreases healthcare costs. Equally, well-timed CBCT investigations can make early diagnostic and accurate treatment decisions possible, potentially decreasing the number of unnecessary visits to oral healthcare specialists and preventing avoidable procedures due to a lack of information [43].

Furthermore, the agreement analysis demonstrated that quantitative evaluation of periapical lesions yields more reliable results compared to qualitative assessment. This finding highlights the importance of utilizing objective measures for accurate diagnosis and treatment planning in the context of periapical lesions. Assuming that all included individuals are diseased based on the inclusion criteria—considered as the “gold standard” or “baseline truth”—in the study, all three methods were compared. Since there are no healthy individuals included in this study, specificity for all methods is 100%, and accuracy cannot be adequately calculated. The only measure that can be calculated is sensitivity, which has been presented in the results above; sensitivity cannot be generalized due to the study design as there are no healthy individuals for comparison. This comparison highlights the superiority of CBCT over PA X-ray while revealing the limitations of the US method. Further, challenges arise with the US method for certain patients in detection processes, mainly when the examination is conducted from the buccal side and when there is no loss of cortical bone or if the cortical bone plate is thick. Additionally, if the periapical process is primarily localized palatinally or lingually and cortical bone loss is observed in this region, the palatal and lingual side examination is hindered due to transducer placement. However, when a periapical inflammatory process is detected, US can provide measurements with sufficient precision.

In this study, we predominantly examined the maxillary incisors, comprising 68% of the total, while the mandibular incisors accounted for 14% of the examined teeth. In contrast, there was less emphasis on examining the maxillary and mandibular canines and premolars, constituting a combined 18% of the examination (Table 1). This study primarily focused on the maxillary and mandibular incisors due to the prevalence of patients scheduled for assessment before apical surgery with periapical inflammations specifically around the incisor region. The lower frequency of examinations on canines, premolars, and molars can be attributed to various factors, such as the comparatively lower demand for apical surgery in these tooth areas. This could be due to challenges such as limited access to the root structure, thicker cortical plates, and the increased complexity of surgical procedures in those regions.

Within the limitations of the design of this study, US has been demonstrated to be a reliable tool for the diagnosis of periapical lesions in dento-facial region. In this study, US and the PA X-ray showed the presence of periapical pathology in all cases. This is in agreement with a study suggesting that dimensional changes in lesions measured with US and PA are compatible [40]. The findings of this study lead to the conclusion that it has a respectable degree of accuracy for periapical lesion diagnosis. US imaging in skilled hands can even aid in the diagnosis of the histopathological nature of the lesion with 100% agreement [38,45], while PA X-ray cannot. Similar results were found in more recent studies, suggesting that US can be used as a diagnostic and measurement tool in periapical lesion diagnostics [20,22]. 

The choice of imaging modality for periapical lesion diagnostics in dentistry involves careful consideration of the clinical requirements, cost factors, and radiation exposure. US as a diagnostic method has been used in many medical fields. However, in dentistry, it has not been thoroughly explored because of diagnostic limitations in relation to transducer size and shape, which restrict access to intraoral scanning. The integration of US in dental diagnostics can augment the traditional use of periapical radiography by leveraging the specific strengths of each imaging modality. While PA radiographs and CBCT are excellent for visualizing hard tissues like bone and teeth, US imaging can provide real-time images and is particularly useful for differentiating soft tissue structures and evaluating soft tissue pathologies due to its ability to capture the physical properties of tissues, such as echogenicity and vascularity. It is essential to integrate clinical observations and symptoms with ultrasound imaging results for an accurate diagnosis to evaluate periapical lesions. The study confirms that US is a reliable tool for precisely measuring periapical lesions, provided that the inflammation localization is not obstructed by anatomical factors such as the thickness of the cortical bone. A high-frequency ‘hockey stick’ linear transducer allows the diagnostic procedure to take place intraorally. Its use provides a more comfortable and accurate procedure for the investigator and patient. Even though US may not always be as definitive as PA radiography or CBCT in diagnosing the existence of dental pathologies—particularly those involving hard tissues—it can still be valuable in specific clinical situations. For example, when a lesion has already been detected, US can be used to assess the lesion’s size and characteristics such as its borders, contents, and the presence of fluid or solid components. Also, ultrasound may be beneficial in cases where ionizing radiation is to be avoided, such as in pregnant patients or young children; however, this is more common in medical rather than dental settings. Moreover, determining the size of a lesion is essential for monitoring the progression or regression of a pathology, guiding biopsy procedures, and for treatment planning. For instance, understanding the extent of a lesion might influence the decision between conservative management versus more aggressive treatment approaches.

## 5. Conclusions

In conclusion, while US may not replace PA radiography in detecting pathologies, it can provide sufficiently accurate size measurements of periapical lesions and ensure additional information about lesion characteristics, essential for patient management when limiting patient radiation exposure is vital. For other situations, the use of CBCT as the most precise method with the highest radiation exposure should be left for more complicated and difficult cases.

## Figures and Tables

**Table 1:** Distribution of the investigated teeth.

	Incisors N (%)	Canines N (%)	Premolars N (%)	Total N (%)
Mandibular	6 (14%)	0 (0%)	1 (2%)	7 (16%)
Maxillary	29 (68%)	4 (9%)	3 (7%)	36 (84%)
Total	35 (82%)	4 (9%)	4 (9%)	43 (100%)

## Data Availability

Data presented in this study are available on request from the corresponding author.
